# Marginal gap and fracture resistance of CAD/CAM ceramill COMP and cerasmart endocrowns for restoring endodontically treated molars bonded with two adhesive protocols: an *in vitro* study

**DOI:** 10.1080/26415275.2020.1728277

**Published:** 2020-02-25

**Authors:** Israa Atif Kassem, Ibrahim Elsebai Farrag, Samir Mahmoud Zidan, Jylan Fouad ElGuindy, Reham Said Elbasty

**Affiliations:** a Department of Fixed Prosthodontics, Faculty of Oral and Dental Medicine, Cairo University, Cairo, Egypt; b M.D.S., Faculty of Oral and Dental Medicine, Cairo University, Cairo, Egypt

**Keywords:** Endocrown, Ceramill COMP, Cerasmart, CAD\CAM, marginal gap, fracture resistance

## Abstract

Endocrowns represent a conservative and esthetic restorative alternative to full coverage crowns. They can be constructed using various CAD/CAM materials that can provide a modulus of elasticity similar to that of teeth. The ability to use of such materials in composite blocks that can be easily repaired is also an advantage, provided appropriate bonding performance is ensured. This study, therefore, evaluated the marginal gap and fracture resistance of two CAD/CAM endocrown materials using two bonding protocols. Thirty-two mandibular molars were evaluated in two groups based on the material type: a Cerasmart group (GC America Inc; *n* = 16) acting as the control and a Ceramill COMP group (Amann Girrbach, Germany; *n* = 16). These groups were then classified according to the bonding protocol used: a total-etch bonding protocol (*n* = 8) and a self-etch bonding protocol (*n* = 8) implemented using RelyX ultimate adhesive resin cement (3M ESPE). The samples were then subjected to aging by simulating a 1-year thermo-mechanical process. The marginal gap results were statistically insignificant across the material and bonding protocol groups before thermo-mechanical aging. Thermo-mechanical aging significantly reduced the marginal gap distance for Ceramill COMP endocrowns cemented using the total-etch protocol (*p* = 0.002). No statistically significant difference was recorded for the fracture resistance in either the material or bonding protocol groups (*p* ≥ 0.05). Both materials and bonding protocols can, therefore, be used in the posterior region providing conservative treatment, adequate marginal gap and fracture resistance.

## Introduction

Endodontically treated teeth have a higher probability of fracture than vital teeth because of their inherently poor structural integrity, with loss of root and coronal dentin resulting from previous caries and/or tooth preparation.^[^^1^^,^^2^^]^ Ceramics with a high mechanical strength and capable of being acid etched (such as those reinforced with leucite or lithium disilicate), along with an adhesive capacity for adhesive systems and resin cements, have made it easier to restore endodontically treated teeth, without cores and intraradicular posts.^[^^3^^]^ Ceramics are feasible for restoring teeth with extensive coronal damage using onlay and/or overlay restorations and more recently with endocrowns.^[^^4^^]^ Endocrowns can be easily applied, require less clinical time compared with conventional crowns, cheaper because of the fewer steps involved, reduces contact time with patient, and are esthetic as they are constructed from ceramic materials.^[^^5^^]^

Applying computer-aided design/computer-aided manufacturing (CAD/CAM) technology to suitable ceramic blocks have made it possible to obtain accurate restorations very quickly. The composite class of CAD/CAM blocks can be divided into two subclasses depending on their microstructure: materials with dispersed fillers and materials with a polymer-infiltrated ceramic network (PICN).^[^^6^^]^ Cerasmart (CS) is a composite block consisting of a flexible nano-ceramic matrix with an even distribution of nano-ceramic and a composite resin containing 71% silica and barium glass nano-particles by weight, exhibiting a flexural strength of 238 MPa.^[^^7^^]^ Ceramill COMP (CC) is a composite CAD/CAM block consisting of strontium boroaluminosilicate glass (78% by weight) and nanofillers, benzyloctadecyldimethylammonium (BODMA), bisphenol-glycidyl dimethacrylate (Bis-GMA), and urethane dimethacrylate (UDMA), exhibiting a compressive strength of 500 MPa and a flexural strength of 191 MPa.^[^^8^^]^

Resin cements are low-viscosity composite materials with filler distribution and initiator content adjusted to allow for a low film thickness and suitable working and setting times. They are widely used for luting low-strength ceramic and laboratory-processed composite restorations.^[^^9^^]^ Currently, cements can be classified into total-etch, self-etch, and self-adhesive resin cements according to the dental tissue treatment or adhesion strategy.^[^^10^^]^ total-etch systems contain phosphoric acid to pretreat the dental hard tissues before rinsing and subsequent application of an adhesive. Total-etch adhesives are offered as two- or three-step systems, depending on whether the primer and bonding come separate or combined in a single bottle.

Self-etch adhesives contain acidic monomers, which etch and prime the tooth simultaneously, and are available as one- or two-step systems. Both total-etch and self-etch systems form a hybrid layer because the resins impregnate the porous enamel or dentin. The choice between total-etch and self-etch systems is often a matter of personal preference.^[^^11^^]^ However, in general, phosphoric acid creates a more pronounced and retentive etching pattern in enamel. Therefore, total-etch bonding systems are often preferred for indirect restorations and when large areas of enamel are still present. Conversely, self-etch adhesives provide a superior and more predictable bond strength with dentin and are therefore recommended for direct composite resin restorations, particularly when predominantly supported by dentin.^[^^12^^]^

Increasing the marginal discrepancy of a crown causes the cement to dissolve and exposes it to the oral environment, leading to microleakage; moreover, a poor margin adaptation increases plaque retention and changes the composition of the subgingival microflora indicating the onset of gingival disease.^[^^13^^]^ Strength is an important mechanical property that determines the performance of brittle materials.^[^^14^^,^^15^^]^ Several factors influence the fracture resistance including the fabrication technique, type of finish line,^[^^16^^]^ final surface finish, and cementation technique.^[^^17^^]^

The present study evaluated the marginal gap and fracture resistance of two CAD/CAM endocrown restorations, namely Cerasmart and Ceramill COMP, with total-etch and self-etch bonding protocols. The null hypothesis of this study was that there would be no difference in the marginal gap or fracture resistance of the Cerasmart and Ceramill COMP when cemented with either the total-etch or self-etch bonding protocols before and after thermo-mechanical aging. This evaluation is expected to aid studies on reducing the risk of fractures in new restorative materials.^[^^18^^]^

## Materials and methods

### Materials and sample preparation

Table 1 lists the chemical composition and manufacturer and product names of the different materials used in this study. A total of 32 CAD/CAM blocks were divided into two subgroups (16 per group) (*n* = 8). The sample size was calculated using the G power software. A large effect size (*f* = 0.5) was expected. The total sample size of 32 blocks (16 per group) was found to be sufficient with a power of 80% and a significance level of 5%. A total number of 32 freshly extracted human mandibular first molars were collected from periodontally affected patients, after they were extracted. The teeth were cleaned, disinfected (ProSpray C-60; Certol International), inspected under light magnification (Stemi DV4 8.0x; Carl Zeiss MicroImaging, Inc), and radiographed to ensure that they were free from cracks and internal resorption.

**Table 1.** Chemical compositions, manufacturers, and product names of the various materials used in this study.

Product name	Type	Composition	Modulus of elasticity	Manufacturer
Cerasmart	Flexible resin nanoceramic blocks	Flexible nano ceramic matrix with an even distribution of nanoceramic	12.1 MPa	GC America, Inc
Ceramill COMP	Ceramic based composite	Strontium boroaluminosilicate glass 78 % nanofillers, BODMA, Bis-GMA, UDMA. Ceramill COMP corresponds to Creamed AMBARINO High-class blanks	13.8 MPa	Amann Girrbach, Germany
RelyX Ulitmate	Dual cure resin cement	Methacrylate monomers, radiopaque, silanated fillers, radiopaque alkaline (basic) fillers, initiator components, stabilizers, rheological additives, pigments and dark cure activator for Scotchbond universal adhesive	7.7 GPa	3M ESPE, Germany

The remining soft tissues were removed using an ultrasonic scaler (Woodpecker UDS-K Ultrasonic Piezo Scaler), and the teeth were disinfected and then stored in a normal saline solution (0.9% sodium chloride) for one week until testing. The average tooth dimensions were 17 ± 2 mm in root length, 10 ± 2 mm in buccolingual, and 9 ± 2 mm in mesiodistal width. The measurements were taken at the cementoenamel junction level using a digital caliper (Vernier Caliper, GB1, China).

### Randomization

The samples were allocated randomly and numbered from 1 to 32. They were divided using the www.random.org website into two main groups (16 each) and two equal subgroups (8 each). The inclusion criteria were the presence of lower molar, absence of carious lesions, no visible fracture lines in the root, complete root formation, and freshly extracted teeth. The exclusion criteria were history of previous endodontic treatments and presence of cracked teeth, carious teeth, internal and external root resorption, dilacerated roots, and lower wisdom teeth.

### Methodology

The teeth were mounted in epoxy resin blocks during endocrown preparation and testing procedures. The teeth were embedded in the resin up to 2 mm below the CEJ (simulated bone level). A specially designed centralizing device was constructed for an accurate placement of the teeth in the epoxy resin blocks using a custom-made metal-square shaped holder (2.5 cm width× 2.5 cm height × 2.5 cm length).^[^^19^^]^

The teeth were endodontically treated, prepared with a butt joint design to receive the endocrown restorations. They were sectioned perpendicular to the long axis 2 mm coronal to the CEJ, using a super coarse diamond disc and copious water irrigation. The pulp chamber of each tooth was accessed using a round bur, and all the root canals were operated by the same operator using the crown down technique. The preparations of the teeth were performed using a milling machine (AF 30; Nouvag AG, Switzerland) equipped with a tapered diamond-coated stainless-steel bur with a rounded end (G845KR, Edenta AG; Basel, Switzerland) for standardization and a retention cavity extending into the pulp chamber 6 mm from the central groove with an 8° divergence from the walls to avoid thinning of the walls. The coronal part of the gutta-percha material was removed using a small carbide bur to 1 mm below the orifice of each canal, and dentin was then conditioned using a dentin adhesive (Scotchbond Universal; 3 M ESPE), which was applied for 15 s, dried thoroughly for 10 s, and light cured; a thin layer of a flowable resin composite (Filtek Z350 XT Flowable Restorative; 3 M ESPE) was bonded to fill the canals up to the level of the pulp chamber to seal the canal orifices and eliminate undercuts.

**Figure 1.**
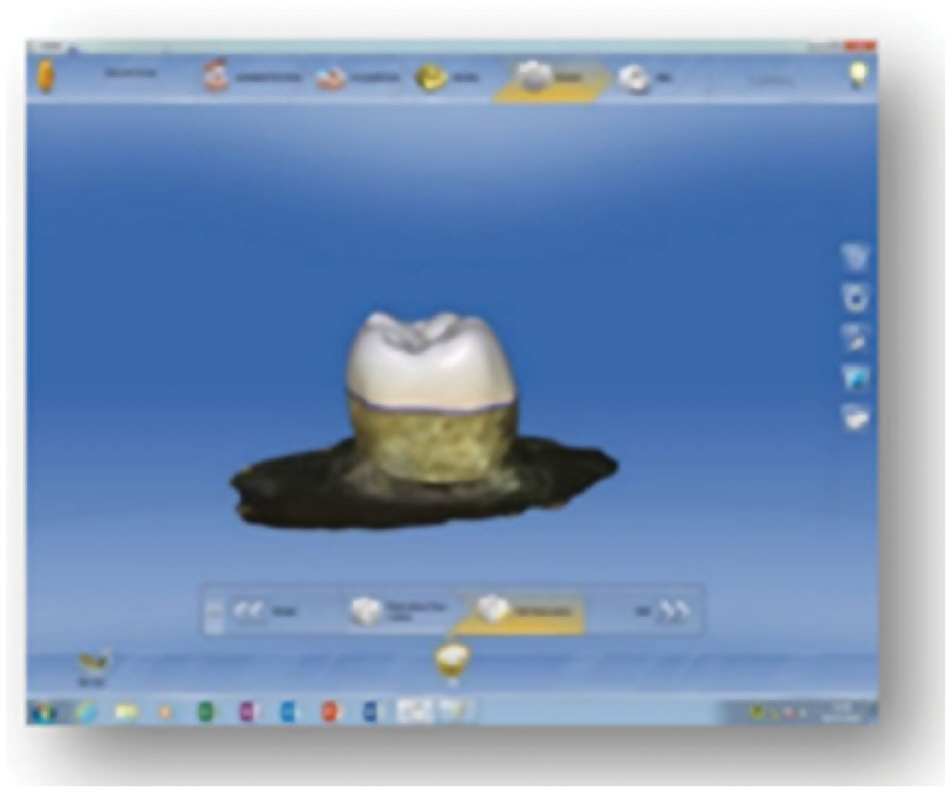
Proximal view of the virtual model for endocrown restoration.

**Figure 2.**
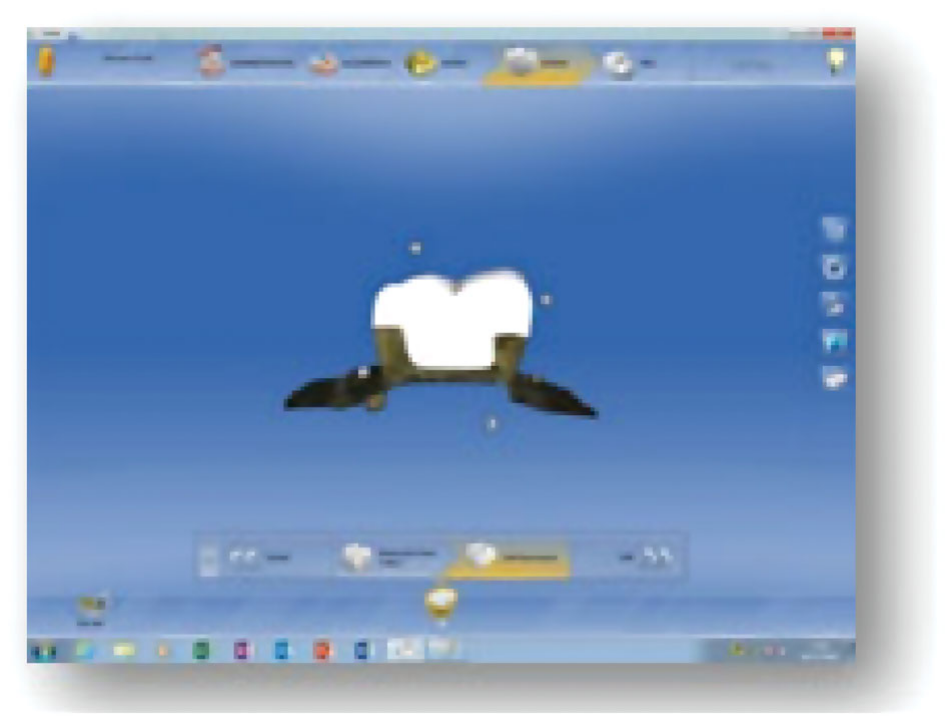
Cross sectional view of virtual model for endocrown restoration.

The teeth were scanned using the CEREC Omnicam, and CEREC software (software 4.4) (Sirona Dental Systems GmbH, Bensheim, Germany) was used to design the restorations. The CEREC MCXL (Sirona Dental Systems GmbH, Bensheim, Germany) machine was used to mill all the restorations, as shown in Figures 1 and 2.

The restorations were finished using a finishing and polishing kit (GC, America Inc) and DiaPolishing paste (GC, America Inc), which was applied with a low-speed hand piece.

### Cementation procedure

#### Surface treatment of Cerasmart endocrowns

The internal fitting surfaces were treated with 5% hydrofluoric acid (DentoBond, Itena Clinical, France) for 60 s, washed under running water, air dried, and applied with a silane coupling agent. Subsequently, ceramic primer II (GC, America Inc) was scrubbed on the fitting surfaces gently, air thinned for 5 s, and left to dry for 1 min; no bonding agent was required after the application of ceramic primer II according to manufacturer’s recommendation. According to the manufacturer’s recommendation, sandblasting or Hydrofluoric acid treatment could be done.

#### Surface treatment of Ceramill^®^ COMP

The Ceramill COMP endocrowns were cleaned in an ultrasonic cleaner (Lk-D32 Codyson CD4820 2.5 L Dental Ultrasonic Cleaner, China) and then gently air dried. The internal fitting surfaces were sandblasted with 25-μm aluminum oxide particles; the sand was removed by an ultrasonic cleaning bath and drying. The surfaces were further cleaned with alcohol according to the manufacturer’s recommendation, and ceramic primer II was gently applied on the fitting surfaces, air thinned for 5 s, and left to dry for 1 min.

#### Surface treatment of the prepared natural tooth

The samples were divided into two groups based on the bonding protocol (total-etch and self-etch). For the total-etch protocol, 35% phosphoric acid (Universal Etchant; 3 M, ESPE, Germany) was applied for 15 s and then rinsed for 20 s. The etched tooth surfaces were gently air dried to avoid over-dried dentin. The prepared teeth were coated to the bonding surface using a Scotchbond Universal Adhesive (3 M, ESPE, Germany) with the help of a micro brush. The adhesive was allowed to dwell for 20 s. It was thinned, and the solvents were evaporated for 5 s with a steady stream of air and then light-cured for 20 s. For the self-etch protocol, the same adhesive was applied directly without the etchant. The adhesive was allowed to dwell for 20 s. It was thinned, and the solvents were evaporated for 5 s with a steady stream of air and then light-cured for 20 s.

The endocrowns were cemented using Rely X Ultimate (3 M Rely X Ultimate; Self-adhesive Cement), as listed in Table 1.

#### Seating of the restorations

Each endocrown was seated on its respective tooth with finger pressure, and excess cement was carefully removed from the margins. A glycerin gel was then applied to all the border surfaces for oxygen inhibition polymerization according to the manufacturer’s instructions.

A customized loading device was then used to apply a constant load of 5 kg parallel to the long axis of each endocrown to prevent rebounding.^[^^19^^]^

### Thermo-mechanical aging

A thermo-mechanical test was conducted using a four-station multi-modal ROBOTA chewing simulator (Model ACH-09075DC-T, Ad-Tech Technology Co., Ltd., Germany), integrated with a thermo-cyclic protocol operated on a servo-motor. The ROBOTA chewing simulator has four chambers simulating the vertical and horizontal movements simultaneously in a thermodynamic condition. The epoxy resin of the samples was readjusted into a cylindrical (3.5 × 3) shape instead of a square shape, to fit the chewing simulator chambers. Each chamber consists of an upper hardened steel stylus holder that can be tightened with a screw for use as antagonistic materials and a lower plastic sample holder in which the specimen can be embedded.

A weight of 10 kg, which is equivalent to a chewing force of 49 N, was exerted. The test was repeated 150,000 times to clinically simulate a one-year chewing condition,^[^^20^^]^ along with thermocycling according to previous studies. The test was conducted at a frequency of 1 Hz in a water bath subjected to a temperature range of 5–55 °C, with an immersion time of 30 s in each dwelling temperature and a drying time of 30 s.^[^^21^^]^

### Marginal gap distance measurement

**Figure 3.**
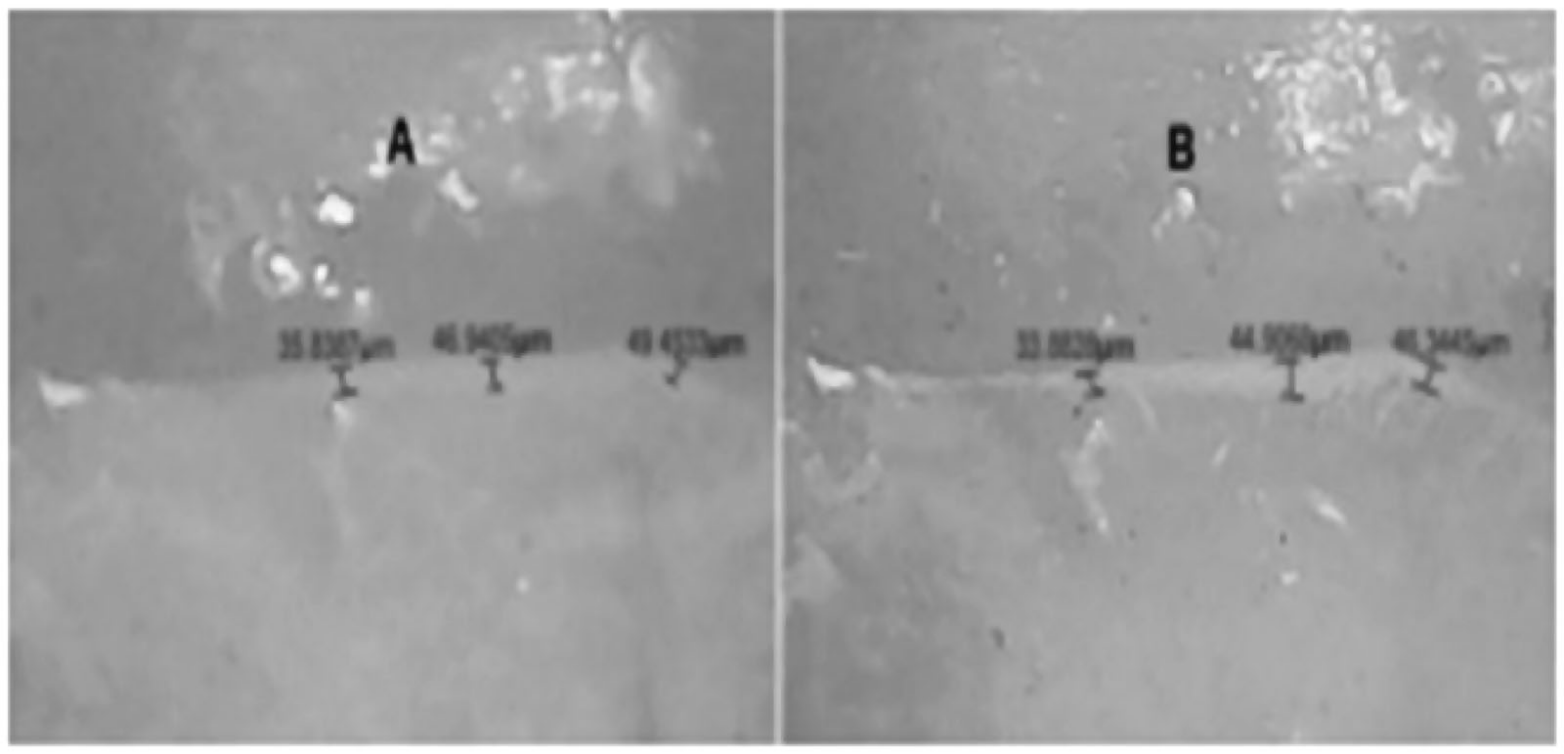
Ceramill COMP steromicroscope (90X magnification) A: before aging; B: after aging.

**Figure 4.**
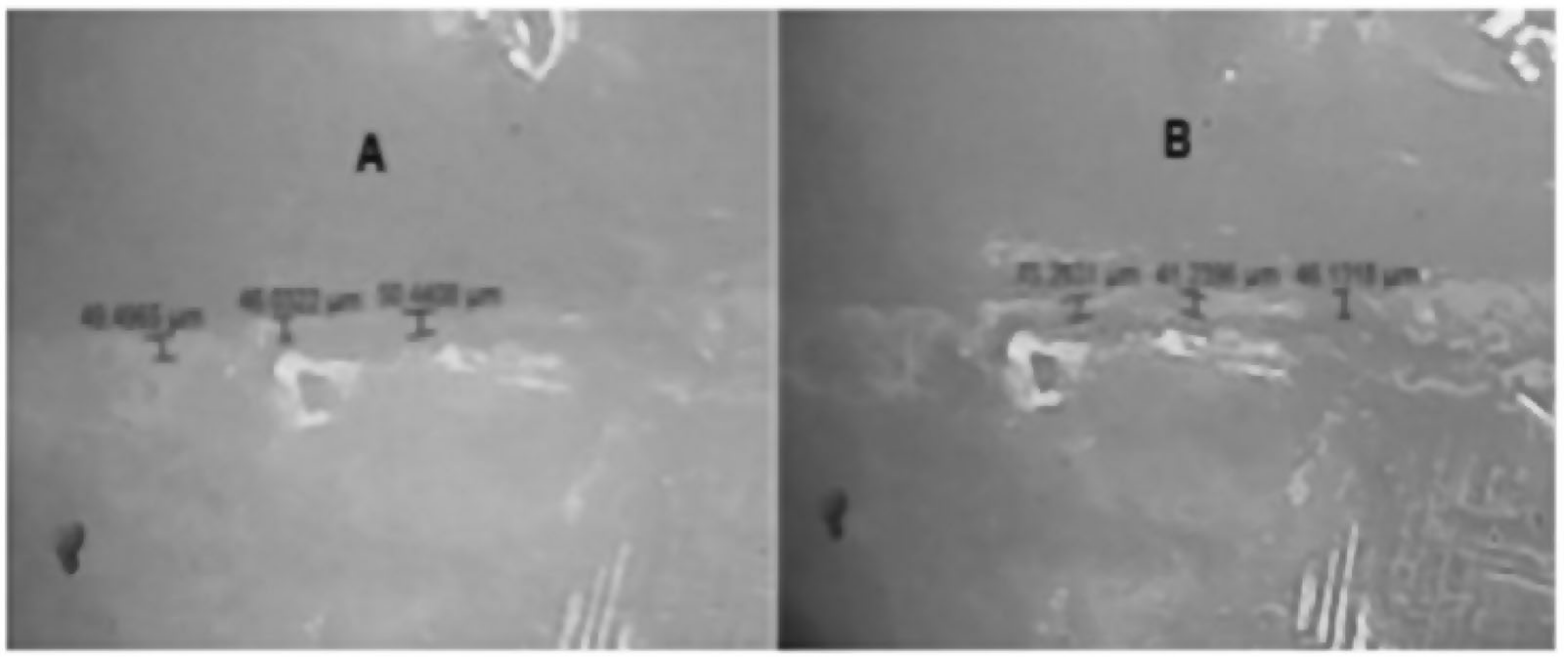
Cerasmart steromicroscope (90X magnification) A: before aging; B: after aging.

The vertical marginal gap distance was evaluated before and after thermo-mechanical aging to detect their effect on the gap distance using a stereomicroscope (Leica S8 APO, German) with a fixed magnification of 90X^[^^22^^]^ (Figures 3 and 4). A digital image analysis system (Image J 1.43 U, National Institute of Health, USA) was used to measure and qualitatively evaluate the gap width. Morphometric measurements were done for each shot (four equidistant landmarks along the cervical circumference for each surface of the specimen (mesial, buccal, distal, and lingual).^[^^19^^]^ The measurement at each point was repeated five times. The images were calibrated each time to ensure the same distance. The data obtained were collected, tabulated, and then subjected to a statistical analysis.

### Fracture resistance testing

After thermo-mechanical aging and marginal gap measurements, the samples were individually mounted on a computer-controlled material testing machine (Model 3345; Instron Industrial) with a loadcell of 5 kN; the data were noted.^[^^23^^]^ The samples were secured to the lower fixed compartment of the testing machine by tightening screws. The fracture test was conducted by applying a compressive load at the center of the occlusal surface in such a way that the load applicator tip only touched the inclined planes of the buccal and lingual cusps, using a metallic rod with a spherical tip (5.6 mm diameter) attached to the upper movable compartment of the testing machine, with a cross-head speed of 1 mm/min.^[^^24^^]^ The load at failure was indicated by a crack and confirmed by a sharp drop on the load–deflection curve recorded using the computer software **(**Bluehill Lite Software Instron^®^ Instruments). The load required to fracture was recorded in Newtons.

### Statistical analysis

The data were collected and coded to facilitate data manipulation and entered to Microsoft Access. The data analysis was performed using the Statistical Package of Social Science (SPSS) software (version 18) on a Windows 7 computer.

A simple descriptive analysis was conducted on the quantitative parametric data in terms of the arithmetic mean as the central tendency measurement and the standard deviation as the measure of the dispersion.

The quantitative data were first tested for normality by conducting a one-sample Kolmogorov–Smirnov test on each study group; the inferential statistic tests were then selected.

For the quantitative data, an independent student *t*-test was used to compare the measures of the two independent groups of quantitative data, and a paired *t*-test was used for comparing two groups of dependent quantitative data. A *p*-value ≤ .05 was considered the cut-off value for significance.

## Results

### Marginal gap before and after thermo-mechanical aging

**Figure 5.**
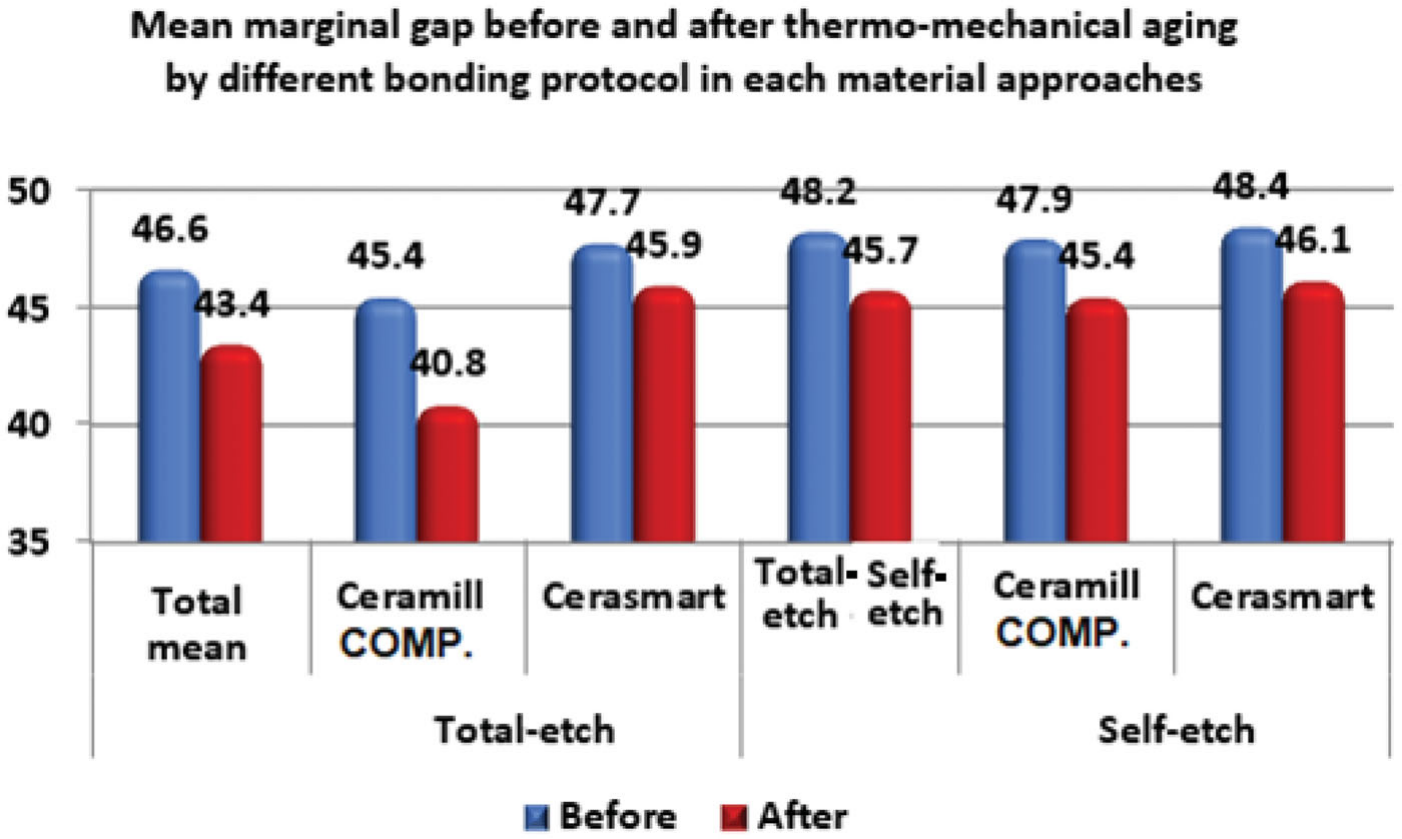
Mean marginal gap before and after thermo-mechanical aging *via* different bonding protocols for each material approach.

The marginal gaps before and after thermo-mechanical are presented in Table 2 and Figure 5. Prior to thermo-mechanical aging, no statistically significant differences in the marginal gap distances of the Cerasmart and Ceramill COMP endocrowns were found for either of the bonding protocols (total-etch: *p* = .3; self-etch: *p* = .7). After exposure to the thermo-mechanical aging process, the Ceramill COMP endocrowns cemented using the total-etch protocol displayed a significantly smaller marginal gap than the Cerasmart crowns (*p* = .002). When both types of endocrowns were cemented using the self-etch protocol, there was no significance difference between the marginal gap resulting from either material (*p* = .06). Thermo-mechanical aging significantly reduced the marginal gap distance for the Ceramill COMP endocrowns that were cemented using the total-etch protocol (*p* = .002), but had no significant influence on the Ceramill COMP crowns cemented using the self-etch protocol (*p* = .2) nor on the Cerasmart crown irrespective of bonding protocol (total-etch: *p* = .3; self-etch: *p* = .08).

**Table 2.** Comparisons of the marginal gaps for the different material groups and bonding protocols.

	Total-etch				Self-etch			
Variables	Mean	Ceramill COMP	Cerasmart	*p*-Value	Mean	Ceramill COMP	Cerasmart	*p*-Value
Before								
Mean	46.6	45.4	47.7	0.3	48.2	47.9	48.4	.7
SD	6.4	5.7	6.9		7.1	6.6	7.8	
After								
Mean	43.4	40.8	45.9	.002***	45.7	45.4	46.1	.06
SD	5.5	6.1	3.2		4.6	5.7	3.2	
*p*-Value	.004*	.002**	0.3		.04*^a^	0.2	0.08	

*Significant difference between the two bonding protocols. **Significant difference between each bonding protocol before and after aging for each material approach. ***Significant difference between the two endocrown materials.

### Fracture resistance

**Figure 6.**
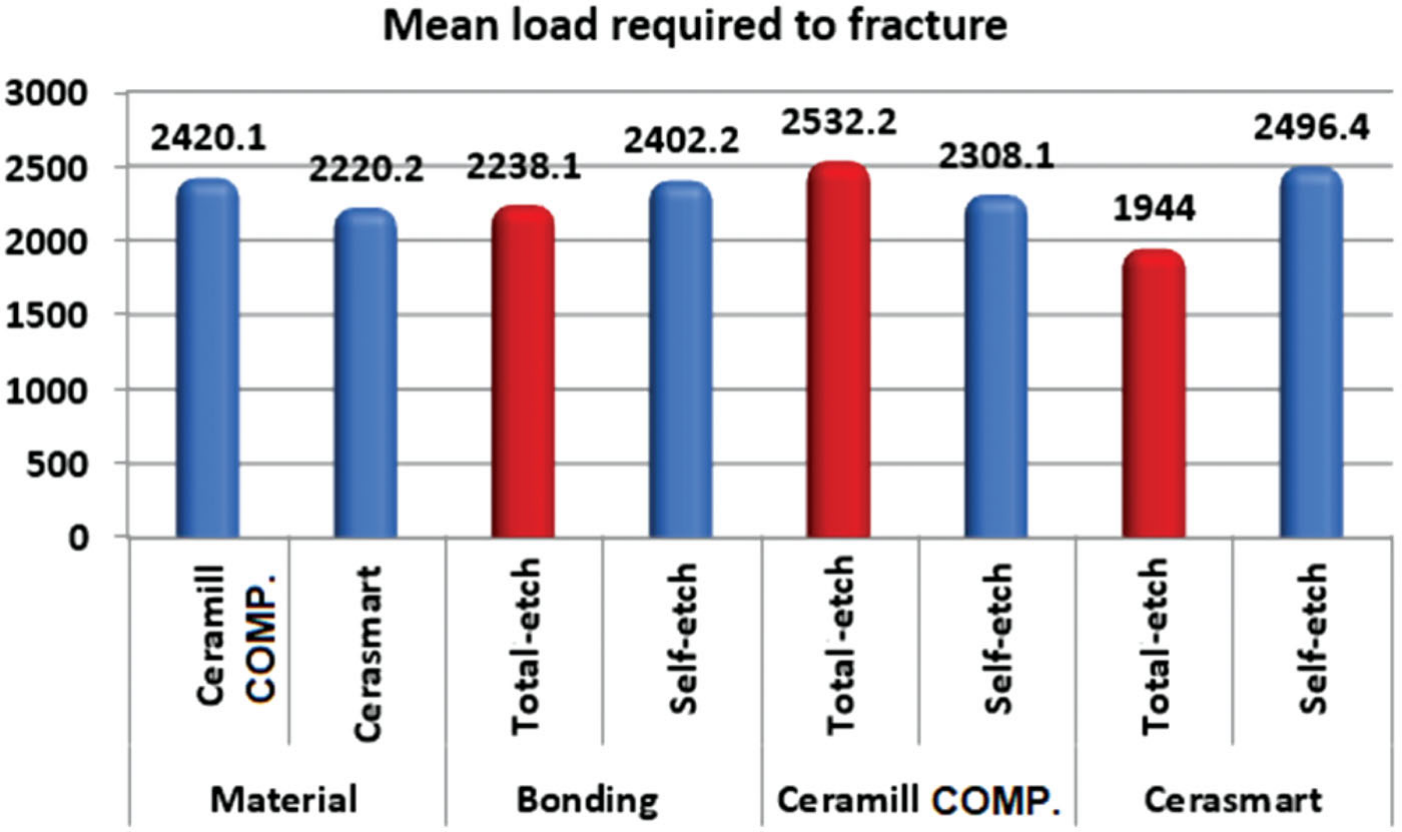
Mean load required to fracture.

With regard to the mean load required to fracture the endocrowns, there were no statistically significant differences between the Ceramill COMP and Cerasmart endocrowns for any of the two bonding protocols (*p* > .05), as shown in Table 3 and Figure 6. Similarly, there were no significant differences between the results obtained for the total-etch and the self-etch bonding protocols for any of the two endocrown materials.

**Table 3.** Comparisons of the load required to fracture the endocrown with respect to the material used and different bonding protocols.

Variables	Mean load required to fracture		*p*-Value
	Mean	SD	
Material cementation approach			
Ceramill COMP	2420	1027	.6
Cerasmart	2220	515	
Bonding protocols			
Total-etch	2238	750	.6
Self-etch	2402	875	
Bonding protocolsin Ceramill COMP cementation			
Total-etch	2532	930	.7
Self-etch	2308	1194	
Bonding protocolsin Cerasmart cementation			
Total-etch	1944	406	.06
Self-etch	2496	487	

## Discussion

Endocrowns take advantage of the recent developments in adhesives, ceramics, and CAD/CAM technology. The utilization of the available space inside the pulp chamber enhances the stability and retention of the restoration and reduces operational errors during post-space preparation.^[^^5^^]^ In-vitro testing was used herein because it overcomes the limitations associated with clinical testing, such as a variation in an individual function, by creating a controlled environment.^[^^25^^]^ Molars were used based on a previous study,^[^^26^^]^ which demonstrated the satisfactory performance of endocrowns for molar teeth in terms of the action of occlusal forces, esthetics, and bond strength. The teeth were prepared according to clinically established preparation criteria for endocrowns.^[^^27^^]^A CEREC Omnicam was used to capture the digital images; this system does not require a reflective medium, which enabled easier and faster image capturing. A CEREC MCXL was used for all the restorations to ensure standardization of the restorations.^[^^28^^]^ Resin blocks Cerasmart and Ceramill^®^ COMP were selected owing to their ability to modify and repair the surface easily and their stress absorbing properties. The structures of the Cerasmart and Ceramill COMP endocrown materials allow for a modulus of elasticity similar to that of dentin (18 ± 2 GPa),^[^^29^^]^ less crack propagation, and higher fracture resistance than conventional ceramics, which are more prone to fracture due to their brittle nature.^[^^30^^]^

The oral cavity is not a static environment. *In vivo*, restorative materials are subjected to dynamic temperature and loading conditions, and *in vitro* simulation of those conditions is essential.^[^^31^^]^ In the present study, mechanical aging accompanied by thermocycling was performed for 150,000 cycles^[^^20^^]^ to simulate a clinical service time of one year.^[^^21^^]^

The most suitable cementation strategy for new restorative materials must be investigated, as a durable bond between the tooth substrate and the restoration is critical to the longevity of the rehabilitation.^[^^32^^]^ Endocrowns were cemented using Rely X Ultimate dual-cure adhesive resin cement. The resin cement bonds to the tooth and to the endocrown through chemical and micromechanical bonding, which provides high retention.^[^^2^^]^ In addition, it acts as an inherent buffering layer capable of absorbing stresses under load, thereby increasing the fracture resistance.

Hydrofluoric acid and silane were used for treating the Cerasmart endocrowns, whereas sandblasting was used to treat the Ceramill COMP endocrowns. The Academy for Adhesive Dentistry reported that hydofluoric acid (HF) etching in combination with silane is a superior pretreatment method for CAD/CAM polymer-infiltrated ceramics.^[^^33^^]^ This recommendation is consistent with the results obtained by Elsaka^[^^34^^]^ and Frankenberger et al.^[^^35^^]^ Sandblasting may cause microcracks in the surface, which may lead to premature failure. It also influences the internal and marginal adaption.^[^^34^^]^ HF leads to micro-porosities on the treated surface, thereby increasing the surface area and enhancing the mechanical interlocking with luting cement; silane acts as a coupling agent between the restoration and the resin bond. The use of HF and silane have been reported as the preferred surface treatment method in several in-vitro studies.^[^^36^^]^

This study found a statistically significant decrease in the mean of the marginal gap width subsequent to thermo-mechanical aging for the Ceramill COMP endocrowns that had been bonded according to the total-etch bonding protocol. The results are in agreement with a study conducted by Elguindy et al.^[^^37^^]^ who concluded that restorations luted with a total-etch system display a lesser marginal gap distance and higher fracture resistance than restorations luted with a self-adhesive system. The decrease in the marginal gap after aging might have been due to the composition of the composites, which are more resilient than ceramics, and this could have affected the stress transferred to the margin walls. Elasticity allows the material to flex while chewing or when under pressure, which in turn would decrease chips, fractures, and stresses acting on the margins.^[^^2^^]^ These results are in accordance with the results obtained by Ramírez-Sebastià et al.^[^^2^^]^ who compared the marginal adaptation of ceramic and composite CEREC endocrowns. The teeth were thermo-mechanically loaded in a chewing simulator. After fatigue, the results showed that thermo-mechanical loading had a significant effect on the marginal gap of both ceramic and composite restorations, where the composite endocrowns fabricated by CEREC exhibited better continuous margins after aging than the ceramic endocrowns.

No change in the statistical significance was found in the mean marginal gap measurements of the Ceramill COMP and Cerasmart endocrowns cemented using the self-etch bonding protocol after thermo-mechanical aging. This could be due to the application of the dentin bonding agent, which generated a well-organized hybrid layer; the self-etch protocol exhibited longer resin tags that result in a continuous resin–dentin bond.^[^^38^^]^ This finding concurs with those of Magne and Douglas,^[^^39^^]^ who showed that dentin bonding agent specimens exhibited a distinct and thicker hybrid zone with more and longer resin tags than the specimens without a dentin bonding agent.

Roperto et al.^[^^40^^]^ and Poggio et al.^[^^41^^]^ also reported that different adhesive strategies significantly affect the bonding of CAD/CAM restorations, composites, or ceramics, and that the self-etch bonding protocol offers better results. Furthermore, it has been argued that the inferior fracture resistance obtained when using the total-etch protocol can be attributed to the incomplete infiltration of the resin monomers into the deeper layers of the dentin once it is demineralized by phosphoric acid and subsequently experiences hydrolytic degradation of the exposed collagen fibrils.^[^^42^^]^ However in this study, no statistically significant difference in fracture resistance was found between the two evaluated bonding protocols. According to the manufacturer’s instructions, RelyX ultimate must be used with a bonding agent. The application of a bonding agent as an intermediate agent can ease the penetration of resin monomers into surface irregularities, allowing for micromechanical interlocking and thus increasing the bond strength.^[^^43^^]^ Despite the consistently promising results of bond strength studies of self-adhesive resin cements on acid-etched enamel, conflicting results describing the efficacy of dentin acid etching on bond strength have been reported.^[^^44^^]^ The results of this study are in agreement with Cruz et al.^[^^45^^]^ who found no difference between the behaviors of the self-etch and total-etch bonding protocols when using different universal adhesives on dentin. They explained this finding by pointing out that the universal adhesives used in their study, including Scotchbond, are classified as mild adhesives because their pH is relatively high, which is why the self-etch and total-etch approaches exhibited no difference in behavior. Muñoz et al.^[^^46^^]^ also reported that Scotchbond universal adhesive was capable of producing similar bond strength to dentin when it was applied using either bonding protocol.

There was no statistical difference between the fracture resistances of the two materials after thermo-mechanical loading. The mean fracture loads recorded in this study were 2420 N for the Ceramill COMP endocrowns and 2220 N for the Cerasmart endocrowns, both of which are far greater than the maximum chewing and biting loads previously reported by Anderson^[^^47^^]^, who measured the loads acting on mandibular molars using strain gauges and found that the maximum whole-tooth load varied between 7.2 and 14.9 kg (70.6 and 146 N) when eating meat, biscuits, or carrots. De Boever et al.^[^^48^^]^ determined that the normal chewing forces exerted on the occlusal surfaces of teeth seldom exceeded 2.4 and 7.2 kg (23.5 and 70.6 N) by using transmitters in a removable fixed partial denture, and concluded that functional chewing forces vary from session to session and with the consistency and viscosity of the food. More recently, the maximum biting forces on the first molar were reported to be approximately 859 N^[^^49^^]^ and 878 N,^[^^50^^]^ whereas the mean maximum bite force varied significantly, ranging from 234 to 597 N in females and from 306 to 847 N in males.^[^^51^^]^ No matter the bite force used, both endocrown materials can clearly accept the applied load without fracture.

Note that the present study did not consider the long-term effects of aging on the marginal gap,^[^^52^^]^ the long-term behavior of restorations,^[^^53^^]^ or the influence of parafunctional habits.^[^^54^^]^ Further research remains required to investigate the longevity of endocrown restorations, particularly under clinical conditions, and the possible influence of parafunction. Successful adhesive bonding can help to increase the fracture resistance of the restored tooth as well as that of the indirect restoration.^[^^55^^]^ It remains a challenge to bond indirect composite restorations to dental hard tissues, because the different interfaces involved (i.e. the interface between the dentin/enamel and adhesive cement and that between the luting agent and the indirect restoration) need to be considered.^[^^43^^]^

As mentioned in the previous discussions and results, this study was designed to assess the vertical marginal gap and fracture resistance of mandibular molars restored using two endocrown CAD/CAM materials, Ceramill COMP and Cerasmart, with two types of bonding protocols, self-etch and total-etch. The null hypothesis was validated in terms of fracture resistance as there was no difference in the endocrowns regardless of their material or bonding protocol used. However, the null hypothesis was rejected in terms of the marginal gap as there was a significant difference in the marginal gap of Ceramill COMP endocrowns cemented by the total-etch bonding protocol after thermo-mechanical aging, compared to the marginal gaps of all other specimen types.

An important limitation of this study was the use of only one type of luting cement system. The use of different luting systems may result in different outcomes. Additionally, the cyclic fatigue was simulated for only one year. Different results may be obtained if the thermo-mechanical aging is simulated for a longer period, such as five years. Further investigation is therefore recommended to continue the study of the vertical marginal gap and fracture resistance of different endocrown materials. Different conditions, such as the use of premolar teeth or different aging parameters, should be used to investigate the same materials under a wider variety of circumstances. Finally, in-vivo studies should be conducted to help predict the clinical success and long-term sustainability of these new endocrown materials.

## Conclusions

The following two main conclusions can be drawn from this study:

- Both Cerasmart and Ceramill COMP are promising endocrown materials in terms of their vertical marginal gap and fracture resistance in the lower posterior region.
- Both the self-etch and total-etch bonding protocols can be used to bond composite endocrowns with adhesive resin cement without concern for long-term behavior over a thermo-mechanical aging period of one year. However, the marginal gap of Ceramill COMP endocrowns cemented by the total-etch bonding protocol was much lower after thermo-mechanical aging than in the other tested specimens.

## Data Availability

All data supporting the reported results are available in a report available from the corresponding author upon request.
